# PRSS23 Promotes Ovarian Follicular Atresia in Wuding Chickens by Coordinately Suppressing Steroidogenesis and PI3K/AKT/mTOR Survival Signaling

**DOI:** 10.3390/genes17030272

**Published:** 2026-02-27

**Authors:** Cailing Wang, Wei Zhu, Enmin Wan, Jinda Li, Xinyang Fan, Yongwang Miao

**Affiliations:** 1 College of Animal Science and Technology, Yunnan Agricultural University, Kunming 650201, China; 18183547507@163.com (C.W.); 15288481209@163.com (W.Z.); 19841354525@163.com (E.W.); 2 Animal Genetics and Breeding Institute, Yunnan Agricultural University, Kunming 650201, China; 3 Institutional Center for Shared Technologies and Facilities of Kunming Institute of Zoology (KIZ), Chinese Academy of Sciences (CAS), Kunming 650223, China; jinda7106@163.com

**Keywords:** Wuding chicken, follicular atresia, *PRSS23*, steroidogenesis, broodiness traits, PI3K/AKT/mTOR

## Abstract

**Background**: Broodiness is a major limiting factor for reproductive efficiency in indigenous avian breeds, a phenomenon underpinned physiologically by granulosa cell (GC) apoptosis and subsequent follicular atresia. While Serine Protease 23 (PRSS23) has been implicated in mammalian ovarian remodeling, its specific regulatory function in avian follicular dynamics remains elusive. **Methods**: Utilizing the Wuding chicken—an indigenous breed distinguished by robust environmental adaptability but compromised by high broodiness frequency—as a biological model, this study dissected the molecular mechanism of PRSS23-mediated follicular regression. We cloned the complete coding sequence of the Wuding chicken *PRSS23* gene, characterized its spatiotemporal expression profile, and interrogated its function in primary GCs via gain- and loss-of-function assays. **Results**: RT-qPCR analysis revealed that *PRSS23* is differentially expressed across the hypothalamic–pituitary–ovarian (HPO) axis, with ovarian expression being significantly upregulated during the broody period compared to the laying period. Mechanistically, *PRSS23* overexpression significantly downregulated the expression of follicle-stimulating hormone receptor (*FSHR*) and key steroidogenic enzymes (*STAR*, *CYP19A1*, *HSD3β1*), thereby suppressing the expression of genes governing the biosynthesis potential of progesterone and estradiol. Concurrently, *PRSS23* overexpression was associated with transcriptional repression of components of the PI3K/AKT/mTOR signaling cascade; this transcriptional regulation further induced cell cycle arrest at the G0/G1 phase, and activated the mitochondrial apoptotic pathway characterized by *BAX* upregulation and *BCL2* downregulation. Conversely, siRNA-mediated knockdown of *PRSS23* alleviated these inhibitory effects, promoting GC proliferation and survival. **Conclusions**: These findings establish PRSS23 as a pivotal pro-atretic factor in Wuding chickens, driving ovarian atrophy through the dual transcriptional-level inhibition of steroidogenesis and survival signaling pathways. This study identifies a potential molecular target for marker-assisted selection programs aimed at attenuating broodiness while preserving the superior meat quality traits of indigenous poultry.

## 1. Introduction

Broodiness acts as an evolutionarily conserved maternal behavior in avian species, ensuring offspring survival through persistent nesting and incubation [1]. However, in the context of modern poultry production, this trait represents a double-edged sword: while essential for natural reproduction, the associated cessation of oviposition severely constrains annual egg production and commercial viability. The Wuding chicken, a renowned indigenous breed from Yunnan Province, China, is prized for its superior organoleptic attributes (e.g., meat texture and flavor) and robust stress resilience. Nevertheless, its industrial scalability is hampered by pronounced broodiness, with hens exhibiting 4–6 broody cycles annually, leading to prolonged reproductive pauses [2]. Consequently, deciphering the molecular mechanisms underlying broodiness is essential for developing effective molecular breeding strategies that balance reproductive efficiency with desirable phenotypic traits.

The physiological manifestation of broodiness involves a profound remodeling of the hypothalamic–pituitary–ovarian (HPO) axis, culminating in ovarian atrophy and the cessation of ovulation [3]. At the cellular level, follicular atresia is the hallmark of this regression, driven primarily by the massive apoptosis of granulosa cells (GCs), which are indispensable for follicular maturation and steroidogenesis [4,5,6]. GC fate is governed by an intricate network of intracellular signals. Specifically, the biosynthesis of steroid hormones, including progesterone (P4) and estradiol (E2), serves not only as a functional output of follicles but also as an autocrine survival signal [7]. Parallelly, the PI3K/AKT/mTOR signaling pathway integrates extracellular cues to promote proliferation and antagonize apoptotic stimuli [8,9]. Although it is currently known that endocrine hormones such as prolactin (PRL) play a pivotal role in inducing broodiness [10], it remains to be further elucidated which key molecules in the local follicular microenvironment trigger GC apoptosis during the onset of broodiness by interfering with steroid synthesis and inhibiting survival signaling pathways.

Serine protease 23 (PRSS23), a highly conserved member of the serine protease family, has emerged as a potential regulator of ovarian function. In murine models, *PRSS23* expression is selectively upregulated in atretic follicles and is suppressed by gonadotropins, suggesting a pro-atretic role [11,12]. Interestingly, in oncology, PRSS23 is often associated with tumor progression and cell proliferation [13,14,15], presenting a functional paradox that implies tissue-specific regulatory mechanisms. However, current research on PRSS23 focuses mainly on mammalian and zebrafish models [16], and its specific function in avian follicular development and atresia, particularly the molecular mechanism in the regulation of broodiness traits, remains largely unknown.

In preliminary transcriptome analyses of Wuding chicken ovaries, we observed a significant upregulation of *PRSS23* during the broody period compared to the laying period [17,18]. Based on this observation, we hypothesized that PRSS23 acts as an active pro-atretic regulator during broodiness by coordinately suppressing steroidogenic capacity and survival signaling in GCs, thereby promoting apoptosis and follicular regression. Elucidating the role of PRSS23 in this process not only advances our understanding of the molecular basis of broodiness but also provides potential genetic targets for the improvement of reproductive performance in indigenous poultry breeds while preserving their valuable adaptive traits.

## 2. Materials and Methods

### 2.1. Ethics Statement

All animal procedures were conducted in strict accordance with the “Regulations for the Administration of Affairs Concerning Experimental Animals” and were approved by the Animal Welfare and Ethics Committee of Yunnan Agricultural University (Approval No. APYNAU202503109).

### 2.2. Experimental Animals and Sample Collection

Wuding chickens were obtained from the breeding farm Yunnan Shouyu Agricultural Development Co., Ltd. in Chuxiong, Yunnan, China. They were housed standard husbandry conditions with ad libitum access to feed and water. Twelve 300-day-old hens were selected and categorized into two groups based on reproductive behavior: the laying group (continuous laying ≥ 5 d, *n* = 6) and the broody group (persistent nesting state ≥ 7 d, *n* = 6). Following euthanasia via cervical dislocation, tissues including the hypothalamus, pituitary, and ovarian stroma were rapidly excised, flash-frozen in liquid nitrogen, and stored at −80 °C for RNA isolation.

### 2.3. Total RNA Extraction, cDNA Synthesis and Gene Cloning

Total RNA was extracted from ovarian stroma tissue of Wuding chickens using the TRIzol method, and the integrity of the RNA was detected by 1.2% agarose gel electrophoresis. cDNA was synthesized using Oligo(dT)_18_ primers (500 μg/mL) and a reverse transcription kit (TaKaRa, Dalian, China). The synthesized cDNA was stored at −20 °C for later use.

Based on the *PRSS23* gene sequence of the chicken in the NCBI database (Accession No.: XM_046904458.1), specific primers were designed using Primer Premier 5.0 to amplify its complete coding sequence (CDS) (primer information is detailed in Table S1). Primers were synthesized by Sangon Biotech (Shanghai) Co., Ltd. Shanghai, China. The PCR amplification system (10 µL) consisted of: upstream and downstream primers at 0.4 µL each (10 µmol/L), 2 × Es Taq Master Mix at 5 µL (CWBIO, Beijing, China), template cDNA at 1 µL (300 ng/µL), and ddH_2_O at 3.2 µL. The PCR amplification program was: 94 °C pre-denaturation for 5 min, followed by 34 cycles of: 94 °C denaturation for 30 s, 58 °C annealing for 30 s, 72 °C extension for 90 s, and finally extension at 72 °C for 7 min. The PCR amplification products were detected by 1.2% agarose gel electrophoresis. The target bands were excised from the gel, purified, and recovered. The recovered products were ligated into the pMD-18T vector (TaKaRa, Dalian, China) and transformed into DH5α competent cells (TransGen Biotech, Beijing, China). Ten positive bacterial clones were selected and sent to Sangon Biotech (Shanghai) Co., Ltd. Shanghai, China, for bidirectional sequencing.

### 2.4. Bioinformatics Analysis

To elucidate the structural characteristics of the *PRSS23* gene transcription region in Wuding chickens, homologous sequences from Phasianidae, Tetraonidae, Anatidae, Psittacidae, Scolopacidae, Muridae, and Hominidae were downloaded from the NCBI database (https://www.ncbi.nlm.nih.gov/genome/?term=, accessed on 9 February 2026) (sequence information is detailed in Table S2) for comparative analysis. Simultaneously, this study utilized multiple bioinformatics tools and databases (detailed in Table S3) to systematically analyze the *PRSS23* across different species, including sequence identity, gene structure, functional domains, protein physicochemical properties, signal peptides, structural prediction, and protein interaction networks.

### 2.5. Isolation and Culture of Wuding Chicken GCs

GCs were isolated from specific hierarchical follicles (F1–F3, diameter > 10 mm) of laying-period Wuding chickens. Briefly, after euthanasia via cervical dislocation, the ovaries were aseptically excised and placed in pre-cooled phosphate-buffered saline (PBS) containing 1% penicillin–streptomycin. Hierarchical follicles were carefully dissected from the ovarian stroma using sterile ophthalmic scissors and forceps, and the follicle surface was gently cleaned to remove adherent connective tissues and blood vessels. Subsequently, the follicles were punctured to release the yolk, and the granulosa layer was carefully stripped off with sterile forceps. The isolated follicular GC layer was washed with PBS, minced into approximately 1 mm^3^ fragments using sterilized surgical scissors, and then centrifuged (1800 r/min, 5 min). The supernatant was discarded, and 10 mL of 0.1% Collagenase Type II (Sigma, St. Louis, MO, USA) and 0.25% Trypsin (Gibco, Grand Island, NY, USA) were added. After mixing uniformly, the mixture was incubated in a 37 °C water bath for 10 min, with gentle shaking every 2 min. After digestion was complete, an equal volume of complete medium was added to terminate digestion. The complete medium consisted of 89% Medium 199 (Gibco), 10% Fetal Bovine Serum (FBS, Gibco), and 1% Penicillin–Streptomycin (Gibco). The suspension was filtered using a disposable 200-mesh sieve and then centrifuged (1800 r/min, 5 min). The supernatant was discarded, and the cells were washed. An equal volume of complete medium was added, and the cells were gently shaken to mix thoroughly before plating. For 6-well cell culture plates, 2 mL of cell suspension was added per well; for 96-well cell culture plates, 100 µL of cell suspension was added per well. After gentle agitation to ensure even distribution, the cell culture plates were placed in a 37 °C, 5% CO_2_ cell culture incubator. After differential attachment for 3 h, the complete medium was changed to remove impurities (fibroblasts/blood cells). Thereafter, the complete medium was changed every 24 h.

### 2.6. Construction of Overexpression Vector, siRNA Synthesis, and Cell Transfection

For overexpression studies, the CDS of *PRSS23* was amplified from Wuding chicken ovarian cDNA using gene-specific primers (Table S1) and subsequently cloned into the pMD-18T vector (TaKaRa). The insert was then excised by double digestion with Kpn I and Hind III, gel-purified, and ligated into the EGFP expression vector (Clontech Laboratories, Palo Alto, CA, USA). The resulting recombinant plasmid, designated EGFP-*PRSS23*, was validated by PCR, restriction enzyme digestion, and Sanger sequencing.

To knockdown the expression of *PRSS23* in chicken ovarian GCs, two specific siRNAs targeting the coding region were designed, siRNA-*PRSS23*-1 (Sense: 5′-GGAAGGACUUCUUGUUGAA-3′; Antisense: 5′-UUCAACAAGAAGUCCUUCC-3′) and siRNA-*PRSS23*-2 (Sense: 5′-GGCUAUGACAGCAGGUUUA-3′; Antisense: 5′-UAAACCUGCUGUCAUAGCC-3′), along with a non-specific negative control siRNA-NC (Sense: 5′-UUCUCCGAACGUGUCACGUTT-3′; Antisense: 5′-ACGUGACACGUUCGGAGAATT-3′). These were synthesized by Sangon Biotech (Shanghai) Co., Ltd. Shanghai, China. To minimize potential off-target effects, two independent siRNAs targeting *PRSS23* were initially tested, and the one with higher knockdown efficiency was selected for subsequent functional analyses.

When cell confluence reached 70–80%, GCs were separately transfected with EGFP-*PRSS23* (500 ng), siRNA-*PRSS23* (60 nM), or their respective controls (EGFP and siRNA-NC) using Lipofectamine 2000 (Invitrogen, Carlsbad, CA, USA) following the manufacturer’s protocol. Each experimental and control group was performed in triplicate. Cells were harvested 48 h post-transfection for subsequent experiments.

### 2.7. Real-Time Fluorescence Quantitative PCR (RT-qPCR)

Total RNA was extracted from the hypothalamic, pituitary and ovarian stroma tissue samples as well as GCs of Wuding chickens using the TRIzol method, and the RNA integrity was examined by 1.2% agarose gel electrophoresis. The purity and concentration of total RNA were determined using a UV–visible spectrophotometer (ALL SHENG, Hangzhou, China). cDNA was synthesized using Oligo(dT)_18_ primers and a reverse transcription kit (TaKaRa, Dalian, China).

The primer sequences used for RT-qPCR in this study are detailed in Table S1, and chicken *GAPDH* (GenBank accession number: NM_205518.2) was used as the reference gene. The RT-qPCR reaction system (20 µL) consisted of: upstream and downstream primers at 0.8 µL each, 10 µL Dib^®^ SYBR qPCR Super Mix Plus, 6.4 µL ddH_2_O (Aibisheng Biotechnology, Beijing, China), and 2 µL cDNA template. The RT-qPCR reaction program was carried out according to the manufacturer’s instructions. The specificity of the amplification was confirmed by the presence of a single peak in the melting curve. Each sample was analyzed in triplicate. RT-qPCR data were analyzed using the 2^−ΔΔCt^ method, and results were compared with the control group.

### 2.8. CCK-8 and EdU Proliferation Detection

Follicular GCs were isolated from the preovulatory follicles (F1–F3) of laying Wuding chickens, and then seeded into 96-well plates and 35 mm confocal culture dishes for subsequent culture in a 37 °C incubator. When cell confluence reached 70%, the cells were transfected. To overexpress *PRSS23*, cells were transfected with the EGFP-*PRSS23* or the EGFP. Conversely, for interference experiments, cells were transfected with the siRNA-*PRSS23* or the siRNA-NC. According to the manufacturer’s instructions, at 0 h, 24 h, and 48 h after transfection, 10 µL of CCK-8 enhanced solution (Beyotime, Shanghai, China) was added to each well. After incubating in the incubator for 2 h, the absorbance at 450 nm was measured, and a cell proliferation line graph was plotted. Additionally, an EdU kit (Beyotime, Shanghai, China) was used, incubated with cells 48 h after transfection. Cells were counterstained with DAPI at room temperature in the dark and observed using a confocal microscope (FV1000, Tokyo, Japan). EdU-positive cells were quantitatively analyzed using ImageJ (v.52a, Bethesda, MD, USA).

### 2.9. Flow Cytometry Analysis

Follicular GCs were seeded in 100 mm culture dishes and transfected when cell confluence reached 70%. The cell cycle was analyzed using a Cell Cycle and Apoptosis Analysis Kit (Beyotime, Shanghai, China). After transfection for 48 h, GCs were collected and washed 3 times with pre-cooled PBS. After fixation with 70% pre-cooled ethanol, cells were stained with a mixture of Propidium Iodide (PI) and RNase A for 30 min at 37 °C in the dark. Data were acquired using a BD FACSCelesta flow cytometer (BD Biosciences, San Diego, CA, USA) and analyzed using FlowJo software (v10.0.7, Tree Star, Ashland, OR, USA).

### 2.10. Statistical Analysis

Data are presented as mean ± SEM from *n* = 6 biological replicates (independent birds) for tissue expression experiments and *n* = 3 independent replicates for cell experiments. Normality and homogeneity of variance were assessed prior to statistical analysis. For comparisons between two groups, an unpaired two-tailed Student’s *t*-test was used. For multiple-group comparisons, one-way ANOVA followed by Tukey’s post hoc test was applied. Statistical analyses were performed using GraphPad Prism 9.0, and *p* < 0.05 was considered statistically significant.

## 3. Results

### 3.1. Morphological Observation of Ovarian Tissues in Laying and Broody Wuding Chickens

Morphological observation of the intact excised ovaries of Wuding chickens revealed that structurally intact prehierarchical and hierarchical follicles were present in the ovaries of laying-phase birds, whereas the ovaries of broody-phase birds exhibited marked atrophy with complete atresia of all follicles (Figure S1). These morphological characteristics verify the accuracy and representativeness of the Wuding chickens selected for the present study.

### 3.2. Cloning Results and Bioinformatics Analysis of the PRSS23 Gene

**Figure 1:**
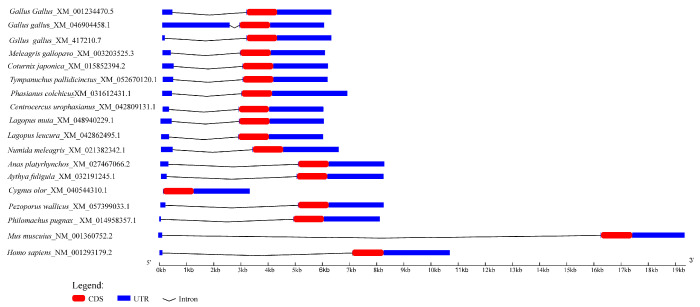
Transcript structures of the *PRSS23* gene in various species.

We successfully cloned the full-length CDS of the *PRSS23* gene from ovarian tissue of broody Wuding chickens (Figure S2). The cloned CDS spanned 1119 bp and encoded a precursor protein of 372 amino acid residues (Figure S3). Genomic structural analysis revealed that the avian *PRSS23* gene shares a conserved bi-exonic architecture with other vertebrate orthologs (Figure 1, Table S4). Multiple sequence alignment demonstrated 100% identity between the Wuding chicken amino acid sequence and the *Gallus gallus* reference sequence (NCBI), and >98.4% identity with other Phasianidae species, highlighting the high evolutionary conservation of this gene (Figure S4).

**Figure 2:**
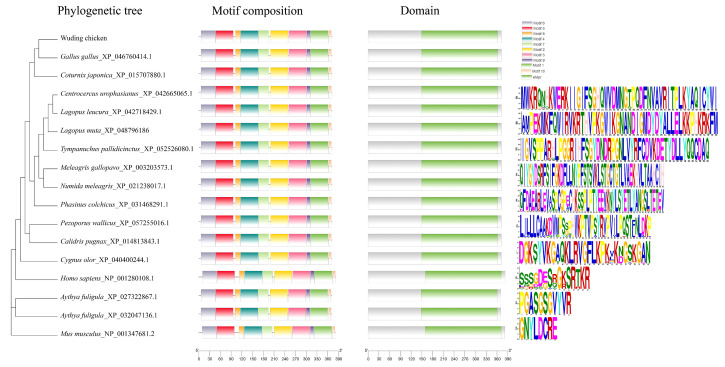
Phylogenetic tree, motif composition and conserved domain of PRSS23 in Wuding chicken and other species. Different amino acids are represented by colored letters.

**Figure 3:**
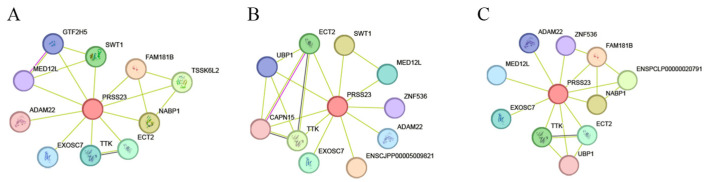
Protein–Protein Interaction (PPI) network of PRSS23 in Wuding chicken (**A**), Japanese quail (**B**) and Ring-necked pheasant (**C**).

Structural motif analysis confirmed the presence of a signature serine protease domain (eMpr). Phylogenetic analysis clustered Wuding chicken PRSS23 into a distinct clade with the Red Junglefowl and Japanese Quail, suggesting a conserved functional role within the Phasianidae family (Figure 2). Physicochemical profiling (Table S5) characterized PRSS23 as a hydrophilic protein. Structural analysis revealed high consistency in secondary/tertiary structures, between Wuding chicken PRSS23 and those of other Phasianidae species (Figure S5, Table S6). Crucially, signal peptide prediction identified a Sec/SPI-type secretory signal sequence at the N-terminus (residues 1–19) and no transmembrane helices in PRSS23, which supports the hypothesis that PRSS23 functions as a secreted serine protease. Furthermore, protein–protein interaction (PPI) network analysis predicted that PRSS23 interacts with key regulators of cell cycle and post-transcriptional control, including ADAM22, EXOSC7, and TTK (Figure 3), implying its involvement in complex cellular regulatory networks.

### 3.3. Spatiotemporal Expression Pattern of PRSS23 in the HPO Axis

**Figure 4:**
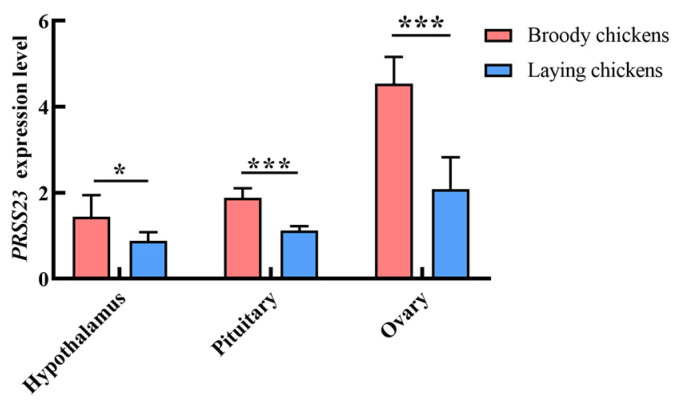
Differential expression of *PRSS23* in the hypothalamic–pituitary–ovarian (HPO) axis of Wuding chickens. The values are presented as means ± SEMs; * *p* < 0.05, *** *p* < 0.001.

To determine the physiological relevance of *PRSS23* to broodiness, we quantified its expression across the HPO axis. *PRSS23* was ubiquitously expressed in the hypothalamus, pituitary, and ovary; however, ovarian tissue exhibited the highest relative abundance (Figure 4). Notably, *PRSS23* expression was state-dependent: mRNA levels in the ovary, pituitary, and hypothalamus were significantly upregulated during the broody period compared to the egg-laying period (*p* < 0.01, Figure 4). The marked ovarian upregulation specifically during broodiness suggests that PRSS23 serves as a local effector driving ovarian regression.

### 3.4. Effect of PRSS23 on the Expression of Steroidogenic Genes

**Figure 5:**
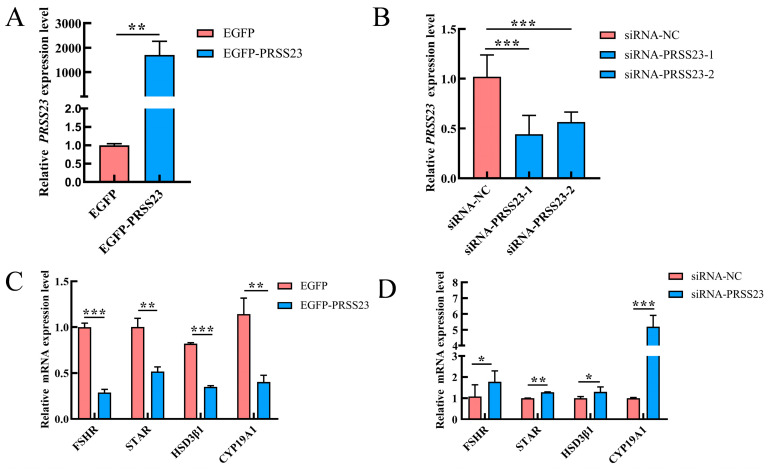
PRSS23 inhibits steroid hormone synthesis in Wuding chicken GCs. Expression levels of the *PRSS23* gene after overexpression (**A**) or interference (**B**). The mRNA expression levels of steroidogenesis-related genes in GCs following *PRSS23* overexpression (**C**) or interference (**D**). The values are presented as means ± SEMs; * *p* < 0.05, ** *p* < 0.01, *** *p* < 0.001.

To further elucidate the role of PRSS23 in regulating the expression of steroidogenesis-related genes in GCs, EGFP-*PRSS23*, EGFP, siRNA-*PRSS23*, and siRNA-NC were transfected into chicken GCs in this experiment. After 48 h, the expression levels of genes related to steroid hormone synthesis in GCs were detected. RT-qPCR analysis showed that the expression level of the *PRSS23* gene in the EGFP-*PRSS23* transfection group was significantly elevated (*p* < 0.01) (Figure 5A), indicating that EGFP-*PRSS23* can be used for subsequent experiments. The expression levels of the *PRSS23* gene were significantly decreased in both the siRNA-*PRSS23*-1 and siRNA-*PRSS23*-2 transfection groups (*p* < 0.001, Figure 5B). As siRNA-*PRSS23*-1 had the best interference effect, siRNA-*PRSS23*-1 was selected for subsequent experiments. Having established the optimal knockdown efficiency, we next assessed the bidirectional functional impact of *PRSS23*. Functional analyses revealed that *PRSS23* overexpression significantly downregulated the expression of key steroidogenesis-related genes, including *FSHR* (*p* < 0.001), *STAR* (*p* < 0.01), *HSD3β1* (*p* < 0.001), and *CYP19A1* (*p* < 0.01) (Figure 5C). Conversely, after interfering with the *PRSS23* gene, the expression levels of the *FSHR*, *STAR*, *HSD3β1*, and *CYP19A1* genes increased significantly (Figure 5D). These data demonstrate that high levels of *PRSS23*, characteristic of the broody state, actively suppress the expression of steroidogenesis-related genes in GCs, likely by desensitizing cells to FSH and blocking downstream steroidogenic enzyme genes.

### 3.5. Effect of PRSS23 Gene on Granulosa Cell Proliferation and Cell Cycle

**Figure 6:**
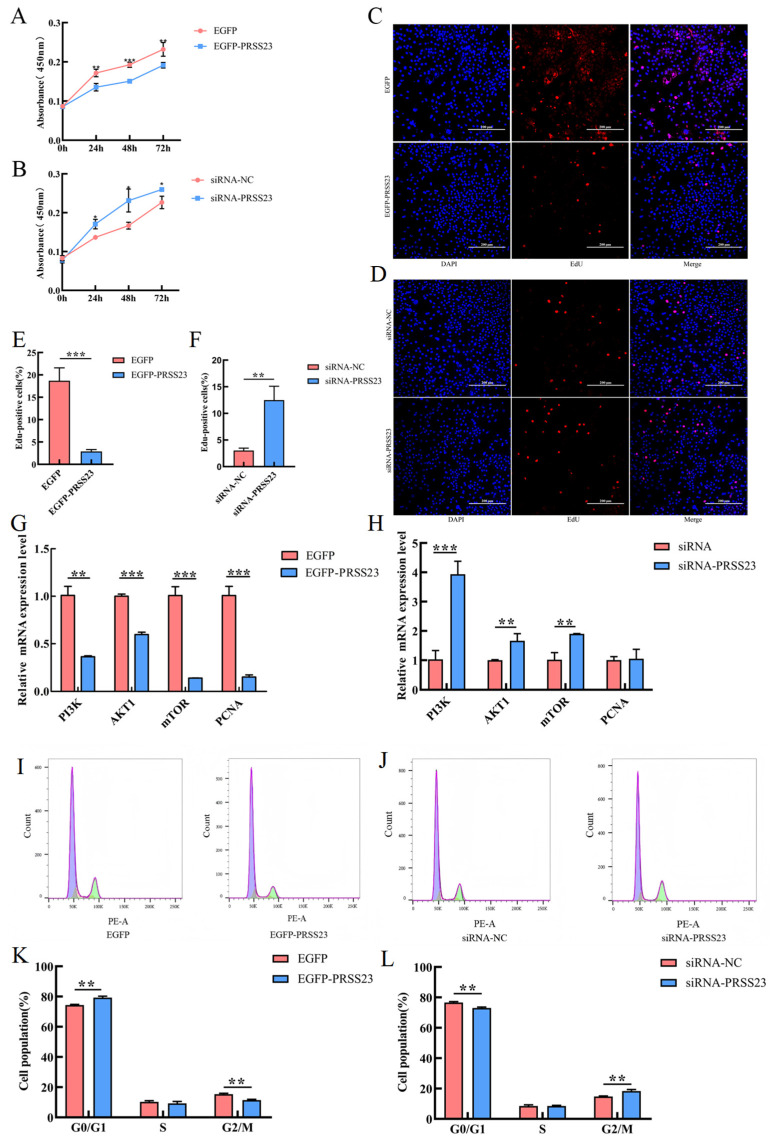
PRSS23 inhibits the proliferation of Wuding chicken GCs. (**A**,**B**) Cell viability of GCs assessed by CCK-8 assay following *PRSS23* overexpression or interference; (**C**–**F**) EdU assay analysis of proliferating GCs after *PRSS23* overexpression or interference. (**G**,**H**) mRNA expression levels of proliferation-related genes in GCs transfected with *PRSS23* overexpression or interference constructs; (**I**,**K**) flow cytometric analysis of cell cycle distribution in GCs after *PRSS23* overexpression. (**J**,**L**) flow cytometric analysis of cell cycle distribution in GCs after *PRSS23* interference. The values are presented as means ± SEMs; * *p* < 0.05, ** *p* < 0.01; *** *p* < 0.001. Note: (**C**,**D**) Red fluorescence marks the proliferating GCs, while blue fluorescence marks the nuclei of the GCs. (**I**,**J**) Representative flow cytometry histograms showing DNA content (PE-A) on the x-axis and cell counts on the y-axis. The first peak from the left represents cells in the G0/G1 phase (2n DNA content), the intermediate region represents the S phase, and the second peak represents the G2/M phase (4n DNA content).

To explore the effect of the *PRSS23* gene on the proliferation and cell cycle of chicken GCs, EGFP-*PRSS23*, EGFP, siRNA-*PRSS23*, and siRNA-NC were transfected into chicken GCs. CCK-8 results showed that overexpression of *PRSS23* significantly reduced granulosa cell viability at 24 h, 48 h, and 72 h after transfection (Figure 6A), while interference with *PRSS23* significantly enhanced granulosa cell viability at 24 h, 48 h, and 72 h after transfection (Figure 6B). EdU staining results showed that the number of positive cells decreased significantly (*p* < 0.001) in GCs transfected with EGFP-*PRSS23* (Figure 6C,E), while it increased significantly (*p* < 0.01) in GCs transfected with siRNA-*PRSS23* (Figure 6D,F). RT-qPCR results showed that overexpression of *PRSS23* was associated with transcriptional repression of components in the PI3K/AKT/mTOR pathway, and significantly downregulated *PI3K* (*p* < 0.01), *AKT1* (*p* < 0.001), *mTOR* (*p* < 0.001), and *PCNA* (*p* < 0.001) (Figure 6G). Conversely, interference with *PRSS23* significantly upregulated the transcription of these genes (Figure 6H). Flow cytometry results are shown in Figure 6I,J. Compared with the control group, the proportion of cells in the G0/G1 phase in the *PRSS23* overexpression group increased significantly (*p* < 0.01), there was no difference in the proportion of cells in the S phase, and the proportion of cells in the G2/M phase decreased significantly (*p* < 0.01) (Figure 6K). In contrast, in the *PRSS23* interference group, the proportion of cells in the G0/G1 phase decreased significantly (*p* < 0.01), there was no difference in the S phase, and the proportion of cells in the G2/M phase increased significantly (*p* < 0.01) (Figure 6L). Collectively, these findings indicate that PRSS23 exerts a suppressive effect on GC proliferation and cell-cycle progression, through the transcriptional downregulation of key genes involved in cell growth and survival signaling.

### 3.6. Effect of PRSS23 Gene on Granulosa Cell Apoptosis

**Figure 7:**
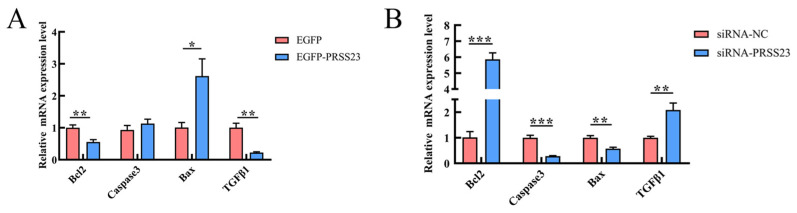
PRSS23 promotes apoptosis in Wuding chicken GCs. Relative mRNA expression levels of apoptosis-related genes in GCs transfected with *PRSS23* overexpression (**A**) or interference (**B**) constructs. The values are presented as means ± SEMs; * *p* < 0.05; ** *p* < 0.01; *** *p* < 0.001.

To explore the effect of the *PRSS23* gene on the apoptosis of chicken GCs, EGFP-*PRSS23*, EGFP, siRNA-*PRSS23*, and siRNA-NC were transfected into chicken GCs. The expression levels of genes related to cell apoptosis in GCs were assessed 48 h after transfection. RT-qPCR results demonstrated that overexpression of the *PRSS23* gene significantly downregulated the mRNA expression levels of anti-apoptotic genes *BCL2* (*p* < 0.01) and *TGFβ1* (*p* < 0.01), while significantly upregulating the mRNA expression level of the pro-apoptotic gene *BAX* (*p* < 0.05) (Figure 7A). In contrast, interference with the *PRSS23* gene significantly upregulated the mRNA expression levels of anti-apoptotic genes *BCL2* (*p* < 0.001) and *TGFβ1* (*p* < 0.01), while significantly downregulating the mRNA expression levels of pro-apoptotic genes *BAX* (*p* < 0.01) and *Caspase3* (*p* < 0.001) (Figure 7B). These results indicate that PRSS23 can promote the apoptosis of chicken GCs.

## 4. Discussion

Broodiness constitutes a fundamental life-history trade-off in avian species, necessitating a dramatic physiological shift from energy-intensive egg production to the behavioral conservation required for incubation. While evolutionarily advantageous for offspring survival in wild populations, this trait manifests as a critical economic bottleneck in the poultry industry, particularly for indigenous breeds like the Wuding chicken which exhibit high-frequency nesting behaviors. The cessation of laying is not merely a behavioral pause but is underpinned by extensive ovarian tissue remodeling, specifically the atresia of hierarchical follicles [3]. Unraveling the molecular hierarchy governing this regression is paramount for reconciling the genetic conflict between resilience and productivity. In the present study, we establish PRSS23 not merely as a biomarker of follicular regression, but as a potent “pro-atretic” mediator that orchestrates follicular degeneration through the coordinate inhibition of steroidogenesis and the PI3K/AKT/mTOR-mediated survival signaling.

Our spatiotemporal analysis reveals a robust upregulation of *PRSS23* in ovarian stromal tissues specifically during the broody phase, inversely correlated with the active laying state. This expression profile mirrors observations in murine models, where *Prss23* and its paralog *Prss35* are characterized as ovarian-selective proteases restricted to atretic follicles [11]. Critically, mammalian studies indicate that *PRSS23* is transcriptionally repressed by gonadotropins; its downregulation by the pre-ovulatory LH surge is a prerequisite for ovulation, whereas its persistence leads to degeneration. In the context of avian broodiness, the endocrine landscape is dominated by elevated PRL, which antagonizes the HPO and suppresses gonadotropin secretion. We propose a conserved regulatory model wherein the withdrawal of gonadotropic support during broodiness lifts the transcriptional repression of *PRSS23*, triggering its aberrant accumulation in the follicular microenvironment. This suggests that *PRSS23* functions as an ancient, vertebrate-conserved “mechanism,” activated specifically when the endocrine command shifts from reproduction to incubation.

A hallmark of healthy follicular development is the capacity of GCs to synthesize steroid hormones. This process is orchestrated by FSH binding to its receptor (FSHR) [19], which activates downstream enzymes such as STAR, CYP11A1, HSD3β1, and CYP19A1. The Steroidogenic acute regulatory protein (STAR) is responsible for the transport of cholesterol to the mitochondria, which is the rate-limiting step in hormone synthesis [20]; Cytochrome P450 cholesterol side-chain cleavage enzyme (CYP11A1) is the key enzyme that converts cholesterol to pregnenolone [21]; 3β-Hydroxysteroid dehydrogenase (HSD3β1) catalyzes the conversion of pregnenolone or dehydroepiandrosterone into bioactive P4 and androstenedione [22]; Cytochrome P450 aromatase (CYP19A1) is the enzyme for the final step of estradiol synthesis [23]. Our functional assays demonstrate that *PRSS23* overexpression significantly downregulated the mRNA levels of *FSHR* and these key steroidogenic genes (*STAR*, *CYP19A1*, *HSD3β1*). The suppression of *FSHR* represents a critical upstream checkpoint. Since *FSHR* signaling via the cAMP/PKA cascade is the primary driver of *CYP19A1* transcription, by downregulating *FSHR*, PRSS23 effectively desensitizes GCs to circulating gonadotropins. This blockade creates a state of localized “hormone resistance” and “hormonal starvation” at the gene expression level, which may underlie the downregulation of estradiol synthesis-related pathways and the subsequent regression of follicular dominance observed in broody hens.

Beyond hormonal desensitization, our study provides the first evidence in avian species that PRSS23 was associated with transcriptional repression of components in the PI3K/AKT/mTOR signaling pathway, the central intracellular arbiter of cell survival and growth [24,25,26]. Although the PI3K/AKT/mTOR pathway is classically modulated via post-translational phosphorylation, our data indicate a crucial layer of regulation wherein PRSS23 significantly represses the transcription of its core components. Our results indicated that *PRSS23* overexpression significantly attenuated the expression of *PI3K*, *AKT1*, and *mTOR*, leading to cell cycle arrest at the G0/G1 phase and the activation of the mitochondrial apoptotic program, and concomitantly suppressed the expression of the proliferation marker gene *PCNA*, which is consistent with the reduced cell viability and decreased number of proliferating cells observed in CCK-8 and EdU assays.

A key mechanistic question arises: How does a secreted serine protease correlate with the transcriptional repression of an intracellular kinase cascade? Given its biochemical function as a secreted protease, PRSS23 may induce anoikis by remodeling the extracellular matrix (ECM). While our current data strongly support this transcriptional outcome, future studies will investigate the direct proteolytic targets of PRSS23 to confirm this ECM-mediated mechanism. GCs are anchorage-dependent; their survival relies on integrin-mediated adhesion to the ECM, which activates Focal Adhesion Kinase (FAK) and subsequently PI3K/AKT. We hypothesize that PRSS23, by proteolytically degrading key ECM components or cleaving extracellular domains of survival receptors, might physically sever the connection between the GC and its microenvironment. Such a loss of “outside-in” signaling could potentially lead to the dephosphorylation of AKT and the collapse of mTOR activity, as observed in our data. This aligns with the protease’s known role in tissue remodeling and suggests that PRSS23 drives atresia by destroying the structural scaffold essential for follicular cell viability, with its transcriptional repression of PI3K/AKT/mTOR pathway components further contributing to this pro-atretic effect. Future studies employing functional assays, such as zymography or ECM marker profiling, will provide additional functional insights into this proposed proteolytic mechanism.

The apoptosis of GC is closely associated with follicular atresia, a process collectively mediated and regulated by the BCL and Caspase families [27,28]. In this study, we found that overexpression of the *PRSS23* gene downregulated the expression of the anti-apoptotic gene *BCL2*, while upregulating the expression of the pro-apoptotic gene *BAX* and the apoptotic effector protein *Caspase3*. The shift in this expression pattern signifies that the comprehensive inhibition of the PI3K/AKT/mTOR pathway disrupts the intracellular balance of BCL2 family proteins and activates the mitochondrial apoptotic pathway, thereby initiating the Caspase3-dependent apoptotic program. This result echoes the findings of Wang [29] in bovine GCs, where PRSS23 acts as a negative regulator limiting cell proliferation capability. Additionally, this study found that PRSS23 can inhibit the expression of *TGFβ1*, which, as a ligand of the TGF-β pathway, promotes cell survival and inhibits apoptosis [30].Therefore, we propose that PRSS23 drives GC apoptosis by inhibiting *TGFβ1* expression, thereby reducing cellular anti-apoptotic potential and synergistically potentiating the activation of the mitochondrial apoptotic cascade. In summary, PRSS23 is likely to exert its pro-apoptotic and anti-proliferative effects on GCs by inhibiting *TGFβ1* expression and disrupts the *BAX/BCL2* balance and subsequently triggers the mitochondrial apoptotic pathway.

These findings highlight a fascinating functional paradox. In human oncology—specifically estrogen-receptor-positive breast cancer and gastric cancer—*PRSS23* acts as an oncogene, promoting proliferation and metastasis. Conversely, in the avian ovary, it acts as a pro-apoptotic factor driving regression. This dichotomy can be resolved by considering the structural context of the tissue. In solid tumors, PRSS23-mediated proteolysis of the ECM clears physical barriers, facilitating cell migration and invasion (EMT). However, in the tightly organized follicular epithelium, the ECM is not a barrier but a lifeline. Its degradation by PRSS23 destroys the spatial cues required for cell polarity and survival, triggering collapse rather than liberation. Furthermore, while PRSS23 is upregulated by estrogen in breast cancer, it appears to be suppressed by the high-estrogen environment of the healthy follicle. This differential regulation underscores PRSS23 as a context-dependent “Janus-like regulator” of tissue remodeling—capable of driving either aggressive expansion or programmed regression depending on the physiological milieu.

Building upon the transcriptional hierarchy delineated in this study, identifying the precise biochemical substrates of PRSS23 in the avian ovary represents an exciting frontier. Additionally, developing specific avian antibodies will enable the corroboration and mapping of these regulatory networks at the translational level.

In summary, this study constructs a comprehensive regulatory model in which an endocrine shift (High PRL/Low FSH) triggers the upregulation of *PRSS23*, which then acts as a “molecular demolition agent” that dismantles steroidogenic capacity, severs PI3K/AKT/mTOR survival signals, activates the mitochondrial apoptotic pathway, and ultimately drives the follicle into programmed atresia.

## 5. Conclusions

This study confirms that PRSS23 is a key effector molecule regulating ovarian functional degeneration during the broody period in Wuding chickens. *PRSS23* exhibits specific high expression in ovarian tissues during broodiness and drives follicular atresia through a dual mechanism. On one hand, it significantly inhibits the expression of *FSHR* and key steroidogenic genes *(STAR*, *CYP19A1*, *HSD3β1*), thereby inhibiting the expression of genes associated with progesterone and estradiol biosynthesis. On the other hand, it disrupts cell survival signals by transcriptionally inhibiting core components of the PI3K/AKT/mTOR signaling pathway, leading to GC cycle arrest and activating the *BAX*/*Caspase3*-mediated mitochondrial apoptosis program. These findings establish the pro-apoptotic status of PRSS23 in avian follicular atresia, providing a new perspective for analyzing the molecular regulatory network of poultry broody behavior and offering a valuable molecular target for the genetic improvement of reproductive performance in local chicken breeds.

## Data Availability

The original contributions presented in this study are included in the article. For further inquiries, please contact the corresponding authors.

## Supplementary Materials

The following supporting information can be downloaded at: https://www.mdpi.com/article/10.3390/genes17030272/s1. Table S1: The information of the primers utilized in this study; Table S2: Nucleotide and amino acid sequence information of PRSS23 across different species; Table S3: The websites of bioinformatics analysis; Table S4: Structural information of PRSS23 transcriptional region in various species; Table S5: Basic physicochemical characteristics of PRSS23 protein in various species; Table S6: Secondary structure composition of PRSS23 proteins in various species; Figure S1: Observation of Ovarian Developmental Phenotype; Figure S2: PCR results of Wuding chicken amplified PRSS23; Figure S3: The CDS and the amino acid sequences encoded by the PRSS23 of Wuding chicken obtained; Figure S4: Amino acid sequence identity of PRSS23 between Wuding chicken and other species; Figure S5: The tertiary structure of PRSS23 mature peptides in Wuding chicken and other pheasant species.

## Abbreviations

The following abbreviations are used in this manuscript:

PRSS23	Serine Protease 23
FSH	Follicle-Stimulating Hormone
FSHR	Follicle-Stimulating Hormone Receptor
STAR	Steroidogenic Acute Regulatory Protein
CYP11A1	Cytochrome P450 cholesterol side-chain cleavage enzyme
HSD3β1	3β-Hydroxysteroid dehydrogenase
CYP19A1	Cytochrome P450 Family 19 Subfamily A Member 1
PI3K	Phosphatidylinositol 3-Kinase
AKT1	AKT Serine/Threonine Kinase 1
mTOR	Mammalian Target of Rapamycin
PCNA	Proliferating Cell Nuclear Antigen
BAX	BCL2-Associated X Protein
BCL2	B-Cell Lymphoma 2
TGFβ1	Transforming Growth Factor Beta 1
Caspase3	Cysteine-Aspartic Acid Protease 3
GAPDH	Glyceraldehyde-3-Phosphate Dehydrogenase
HPO	Hypothalamic–Pituitary–Ovarian Axis
GCs	Granulosa Cells
ECM	Extracellular matrix
P4	Progesterone
E2	Estradiol
RT-qPCR	Real-Time Fluorescence Quantitative PCR
cDNA	Complementary Deoxyribonucleic Acid
siRNA	Small interfering RNA
SiRNA-NC	Small interfering RNA Negative Control
PI	Propidium Iodide
EGFP	pEGFP-N1
CCK-8	Cell Counting Kit-8
EdU	5-Ethynyl-2′-deoxyuridine
CDS	Coding DNA Sequence
