# Supplementary material for: PRSS23 Promotes Ovarian Follicular Atresia in Wuding Chickens by Coordinately Suppressing Steroidogenesis and PI3K/AKT/mTOR Survival Signaling
Source: Genes (Basel). 2026 Feb 27;17(3):272. doi: 10.3390/genes17030272 (PMC13026264; doi:10.3390/genes17030272)
Supplement: Supplementary file 1 [file genes-17-00272-s001.zip › genes-4144595-supplementary.pdf]

Supplementary Materials

Table S1. The information of the primers utilized in this study

Primer ID	Primer sequence(5′→3′)	Products length	Annealing temperature /℃	Usage
PRSS23	F:AGAATGGCTGGCTTGACC<br>R:ACAAGGCGGCTCACTTGA	1119	58	Gene isolation
PRSS23	F:TATCCTGCCACAGTCTACCT<br>R:ACAAGGCGGCTCACTTGA	243	59.5	Expression detection
GAPDH	F:ATCTTCCAGGAGCGTGACC<br>R:CACGATGCATTGCTGACAA	230	60	Expression detection
PRSS23	F:AAGCTTATGGCTGGCTTGACCACTCTA<br>R:GGTACCTCCTTCCCTGCAGTCAAGATAGT	1119	60	Construction of recombinant vector
FSHR	F:GAGCGAGGTCTACATACAAG<br>R:CGCATGCCATAATGGGAAAT	241	61.5	Expression detection
STAR	F:TGCCATCTCCTACCAACA<br>R:CATCTCCATCTCGCTGAAG	190	60	Expression detection
HSD3β1	F:GCCAAAGAGGAGCAAACCAGAG<br>R:CATCTCCATCTCGCTGAAG	104	60	Expression detection
CYP19A1	F:ATGTTCCATCACGCTATTT<br>R:GATTCTTGTTTGGGCTTCT	240	60	Expression detection
PI3K	F:CAGGTGGAGGCTATGGAGAAG<br>R:TGCACTCCTCAAGCCGAAG	196	57.5	Expression detection
AKT1	F:TGATGGCACATTCATTGGCTAC<br>R:TGTTTGGTTTAGGTCGTTCTGTCT	122	57.5	Expression detection
mTOR	F:GCACAAACCTCTGCGACAT<br>R:CGTGAGCAAGCGAGAACAA	250	62	Expression detection
PCNA	F:AGCACCAAATCAGGAAAAG<br>R:GCACAGGAGATGACAACAG	177	60	Expression detection
BCL2	F:CAGAGGGACTTCGCCCAGAT<br>R:TGTGCAGGTGCCGGTTCAG	235	60	Expression detection
Caspase3	F:AAAAGATGGACCACGCTCAG<br>R:CTCGGTGGAAGTTCTTATTG	182	61.5	Expression detection
TGFB1	F:GCCGTCCATCAGTGGCTGAG<br>R:GGTGCTTCTTGGCAATGCTCTG	163	61.5	Expression detection
BAX	F:ACTCTGCTGCTGCTCTCCTCTC<br>R:CCGCTCTCTGCCTTCTCAATGATG	143	62	Expression detection

Tab S2 Nucleotide and amino acid sequence information of PRSS23 across different species

species	Taxonomic Family	Accession number of nucleotide sequences	Accession number of protein sequences
Gallus gallus	*Phasianidae*	XM_046904458.1	XP_046760414.1
Meleagris gallopavo	*Phasianidae*	XM_003203525.3	XP_003203573.1
Coturnix japonica	*Phasianidae*	XM_015852394.2	XP_015707880.1
Phasianus colchicus	*Phasianidae*	XM_031612431.1	XP_031468291.1
Tympanuchus pallidicinctus	*Phasianidae*	XM_052670120.1	XP_052526080.1
Centrocercus urophasianus	*Tetraonidae*	XM_042809131.1	XP_042665065.1
Lagopus muta	*Tetraonidae*	XM_048940229.1	XP_048796186
Lagopus leucura	*Tetraonidae*	XM_042862495.1	XP_042718429.1
Numida meleagris	*Numididae*	XM_021382342.1	XP_021238017.1
Anas platyrhynchos	*Anatidae*	XM_027467066.2	XP_027322867.1
Aythya fuligula	*Anatidae*	XM_032191245.1	XP_032047136.1
Cygnus olor	*Anatidae*	XM_040544310.1	XP_040400244.1

Pezoporus wallicus	*Psittaculidae*	XM_057399033.1	XP_057255016.1
Calidris pugnax	*Scolopacidae*	XM_014958357.1	XP_014813843.1
Mus musculus	*Muridae*	NM_001360752.2	NP_001347681.2
Homo sapiens	*Hominidae*	NM_001293179.2	NP_001280108.1

Tab S3 The websites of bioinformatics analysis

Software and Tools	Analysis content	Online website
NCBI	Download homologous sequences	https://www.ncbi.nlm.nih.gov/
ORF Finder	identify and determine the open reading frame	https://www. nebi. nlm. nih. gov/orffinder/
Gene Structure Display Server	Transcriptional region structure analysis	https://gsds.gao-lab.org/
MEME Suite	Motif	https://meme-suite.org/meme/
Expasy-ProtParam	Physicochemical Properties	https://web. expasy. org/protparam
Expasy-ProtScale	Hydrophilicity prediction	https://web. expasy. org/protscale
SignalP 6.0 Server	Signal peptide prediction	https://services.healthtech.dtu.dk/services/SignalP-6.0/
TMHMM 2.0 Server	Transmembrane domain prediction	https://services.healthtech.dtu.dk/services/TMHMM-2.0/
PROSITE	Post-translational modification site predictio n	https://prosite.expasy.org/
PSORT Ⅱ	Subcellular localization prediction of proteins	https://www. genscript. com/psort. html
PSIPRED	Prediction of protein secondary structure	http://bioinf.cs.ucl.ac.uk/psipred/
SWISS-MODEL	Tertiary structure prediction	https://swissmodel. expasy. org/interactive
STRING12.0	Protein-protein interaction prediction	https://version-12-0.string-db.org/cgi/input?sessionId=bxLIk2v3a7iB&input_page_show_search=on

Tab S4 Structural information of PRSS23 transcriptional region in various species

species	number of exon	number of intron	Length(bp)				
			5′UTR	E1	E2	CDS	3'UTR
Gallus gallus XM_001234470.5	2	1	389	376	3124	1119	1992
Gallus gallus XM_046904458.1	2	1	2497	2484	3124	1119	1992
Gallus gallus XM_417210.7	2	1	109	96	3124	1119	1992
Meleagris gallopavo XM_003203525.3	2	1	316	303	3130	1119	1998
Coturnix japonica XM_015852394.2	2	1	434	421	3147	1119	2015
Phasianus colchicus XM_031612431.1	2	1	370	357	3904	1119	2772
Tympanuchus pallidicinctus XM_052670120.1	2	1	407	394	3126	1119	1994
Centrocercus urophasianus XM_042809131.1	2	1	200	239	3131	1119	1999
Lagopus muta XM_048940229.1	2	1	430	417	3128	1119	1996
Lagopus leucura XM_042862495.1	2	1	303	290	3130	1119	1998
Numida meleagris XM_021382342.1	2	1	440	427	3177	1119	2045
Anas platyrhynchos XM_027467066.2	2	1	320	200	3177	1116	2048
Aythya fuligula XM_032191245.1	2	1	222	209	3199	1116	2070
Cygnus olor XM_040544310.1	2	1	35	22	3197	1119	2065
Pezoporus wallicus XM_057399033.1	2	1	199	160	3160	1119	2028
Calidris pugnax XM_014958357.1	2	1	77	64	3188	1119	2056
Mus musculus NM_001360752.2	2	1	161	150	3091	1149	1931
Homo sapiens NM_001293179.2	2	1	118	110	3598	1152	2438

Table S5 Basic physicochemical characteristics of PRSS23 protein in various species

Species	Amino·acid number	pI	Relative· molecular weight （KDa）	Instability index	GRAVY	number of positively charged residues	number of negatively charged residues	number of hydrophobic amino acids	number of polar amino acids
Wuding chicken	372	9.40	42.00	41.45	-0.494	52	34	110	159
Gallus gallus	372	9.40	42.00	41.45	-0.494	52	34	110	159
Meleagris gallopavo	372	9.37	41.96	40.42	-0.471	52	34	111	101
Coturnix japonica	372	9.39	41.96	41.02	-0.479	52	34	111	101
Phasianus colchicus	372	9.40	41.98	41.25	-0.481	52	34	111	101
Tympanuchus pallidicinctus	372	9.40	41.96	40.81	-0.462	52	34	113	99
Centrocercus urophasianus	372	9.40	42.00	41.04	-0.483	52	34	111	102
Lagopus muta	372	9.40	41.94	40.29	-0.476	52	34	112	100
Lagopus leucura	372	9.40	41.94	40.29	-0.476	52	34	112	100
Numida meleagris	372	9.39	42.15	36.75	-0.506	53	35	111	100
Anas platyrhynchos	371	9.40	41.82	40.84	-0.456	51	33	112	102
Aythya fuligula	371	9.40	41.82	40.84	-0.456	51	33	112	102
Cygnus olo	372	9.40	41.87	40.75	-0.456	51	33	112	102
Pezoporus wallicus	372	9.35	41.90	39.57	-0.461	51	34	112	102
Calidris pugnax	372	9.40	42.03	37.25	-0.491	52	34	110	102
Mus musculus	382	9.54	43.07	37.29	-0.478	55	33	115	103
Homo sapiens	383	9.48	43.00	36.05	-0.478	52	32	115	107

Table S6 Secondary structure composition of PRSS23 proteins in various species

Species	α-Helix	Extended Strand	BetaTurn	Random Coil
Wuding chicken	15.05	25.00	5.28	54.57
Gallus gallus XP_046760414.1	15.05	25.00	5.28	54.57
Meleagris gallopavo XP_003203573.1	16.94	23.66	4.30	55.11
Coturnix japonica XP_015707880.1	14.78	24.46	4.30	56.45
Phasianus colchicus XP_031468291.1	15.32	24.46	4.03	56.18
Tympanuchus pallidicinctus XP_052526080.1	15.05	24.46	4.03	56.45
Centrocercus urophasianus XP_042665065.1	13.98	24.73	3.49	57.80
Lagopus muta XP_048796186	13.98	25.54	4.30	56.18
Lagopus leucura XP_042718429.1	13.98	25.54	4.30	56.18
Numida meleagris XP_021238017.1	17.20	23.92	3.76	55.11
Anas platyrhynchos XP_027322867.1	13.75	26.15	4.58	55.53
Aythya fuligula XP_032047136.1	13.75	26.15	4.58	55.53
Cygnus olor XP_040400244.1	15.32	25.27	4.84	54.57
Pezoporus wallicus XP_057255016.1	15.05	23.66	3.49	57.80
Calidris pugnax XP_014813843.1	14.25	25.00	3.49	57.26
Mus musculus NP_001347681.2	15.97	24.08	4.45	55.50
Homo sapiens NP_001280108.1	14.62	23.50	4.96	56.92

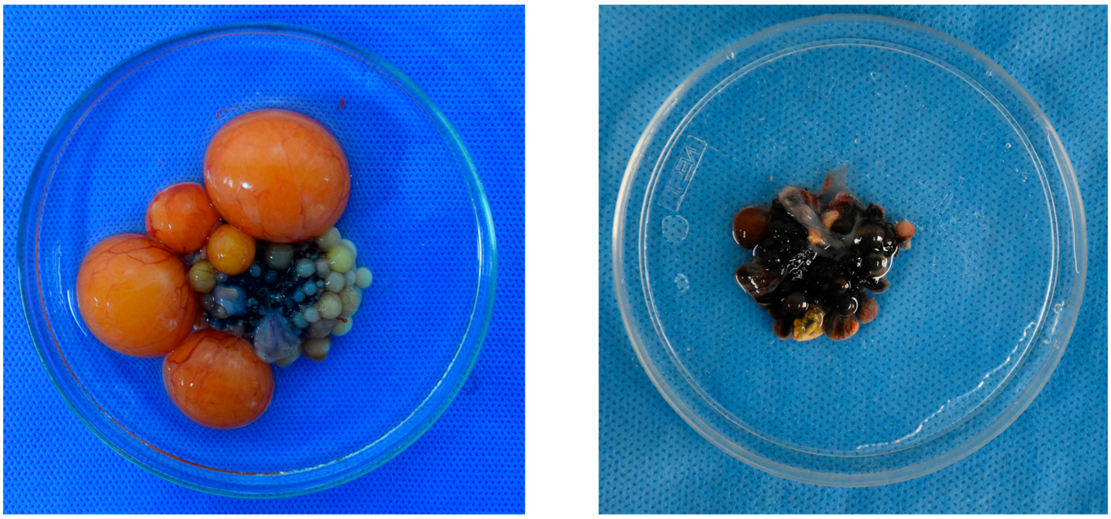

laying period　　broody period

Figure S1: Observation of Ovarian Developmental Phenotype

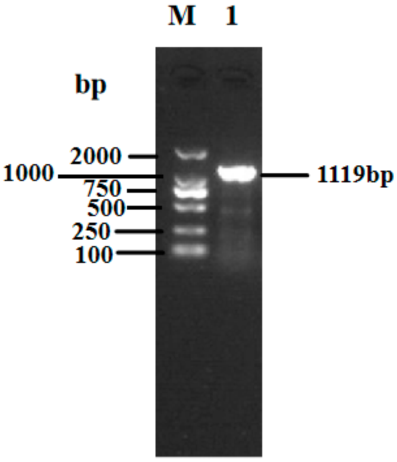

Figure S2: PCR results of Wuding chicken amplified PRSS23

```
   1 ATGGCTGGCTTGACCACTCTAATCCTCCTTTTGTGTGCTGCTAAGGATGTGATGCCCTCCAGTCCTCACTGGAAGCCAACTTGGCCCTCG 90
   1 M  A  G  L  T  T  L  I  L  L  L  C  A  A  K  D  V  M  P  S  S  P  H  W  K  P  T  W  P  S   30
  91 TACAGAGTTCCAGTTATCCTGCCACAGTCTACCTTTCACTTGGACAAACCACAGTTCGATGCTGAAGCCAGACTGGAAGTGGCATCTCCG 180
  31 Y  R  V  P  V  I  L  P  Q  S  T  F  H  L  D  K  P  Q  F  D  A  E  A  R  L  E  V  A  S  P   60
 181 GCTCTTCAGTTCGTATGTGGAGACAGAGGCTTCTACTTCAGTAAGCCTACAGGGTATGGATCCAGCAGTAGACGCTTACACCACAAGGGA 270
  61 C  G  P  E  C  H  K  S  S  P  L  P  T  Y  E  E  V  K  N  Y  L  S  Y  E  T  L  Y  A  N  G   90
 271 TGTGGCCCAGAGTGCCACAAAAGTTCTCCGCTTCCAACTTATGAAGAAGTGAAGAACTACCTGTCCTATGAAACCTTGTATGCTAACGGT 360
  91 S  L  T  E  T  E  V  G  I  Y  I  L  S  S  G  G  D  E  S  R  G  K  S  R  T  K  R  Q  I  Y   120
 361 GGCTATGACAGCAGGTTTAGCATTTTTGGGAAGGACTTCTTGTTGAATTACCCGTTCTCCACGTCAGTGAAGCTATCTACAGGTTGCACG 450
 121 G  Y  D  S  R  F  S  I  F  G  K  D  F  L  L  N  Y  P  F  S  T  S  V  K  L  S  T  G  C  T   150
 451 GGGACGCTAGTGGCTGAAAAGCACGTGCTCACCGCTGCTCATTGCATCCACGATGGCAAGAGTTATGTCAAAGGAGCTCAGAAACTGCGG 540
 151 G  T  L  V  A  E  K  H  V  L  T  A  A  H  C  I  H  D  G  K  S  Y  V  K  G  A  Q  K  L  R   180
 541 GTGGGGTTCCTGAAGCCTAGAGGGAAAAATGGCAGCAAAGGGGCCAACATCACCAGCTCAGCAATGCCTGAGAAAATGAAATTCCAGTGG 630
 181 V  G  F  L  K  P  R  G  K  N  G  S  K  G  A  N  I  T  S  S  A  M  P  E  K  M  K  F  Q  W   210
 631 ATCCGGGTGAAACGGACACATGTCCCCAAAGGATGGATCAAAGGCAATGCCAATGATATTGGCATGGATTATGACTATGCCCTGCTGGAG 720
 211 I  R  V  K  R  T  H  V  P  K  G  W  I  K  G  N  A  N  D  I  G  M  D  Y  D  Y  A  L  L  E   240
 721 CTCAAGAAGCCTCATAAAAGAAAGTTTATGAAGATAGGTGTGAGCCCGCCAGCAAGACACTTGCCTGGAGGGAGAATTCACTTTTCTGGC 810
 241 L  K  K  P  H  K  R  K  F  M  K  I  G  V  S  P  P  A  R  H  L  P  G  G  R  I  H  F  S  G   270
 811 TATGACAATGATCGACCAGGAAACCTGGTGTACCGTTTCTGTGACGTCAAAGATGAAACGTACGACCTGTTGTACCAGCAGTGTGATGCC 900
 271 Y  D  N  D  R  P  G  N  L  V  Y  R  F  C  D  V  K  D  E  T  Y  D  L  L  Y  Q  Q  C  D  A   300
 901 CAGCCAGGTGCCAGTGGATCTGGGGTGTACGTGAGGATGTGGAAGAGGCAGAATCACAAATGGGAACGTAAAATTATTGGCATATTTTCA 990
 301 Q  P  G  A  S  G  S  G  V  Y  V  R  M  W  K  R  Q  N  H  K  W  E  R  K  I  I  G  I  F  S   330
 991 GGCCATCAGTGGGTGGACATGAATGGCACCCCGCAGGATTTCAATGTAGCTGTTCGCATCACACCCCTCAAATACGCACAGATCTGTTAC 1080
 331 G  H  Q  W  V  D  M  N  G  T  P  Q  D  F  N  V  A  V  R  I  T  P  L  K  Y  A  Q  I  C  Y   360
1081 TGGATCAAAGGCAACTATCTTGACTGCAGGGAAGGATAA                                                    1119
 361 W  I  K  G  N  Y  L  D  C  R  E  G                                                          372
```

Figure S3: The CDS and the amino acid sequences encoded by the PRSS23 of Wuding chicken obtained

	1	2	3	4	5	6	7	8	9	10	11	12	13	14	15	16	17		
1		100.0	98.1	98.4	98.4	97.3	97.6	97.6	97.6	97.0	96.5	96.5	96.5	96.0	95.7	84.1	86.3	1	Wuding chicken
2	0.0		98.1	98.4	98.4	97.3	97.6	97.6	97.6	97.0	96.5	96.5	96.5	96.0	95.7	84.1	86.3	2	*Gallus gallus* XP_046760414.1
3	1.9	1.9		99.2	99.5	98.9	98.7	99.2	99.2	97.8	97.0	97.0	97.0	96.5	96.5	84.1	86.8	3	*Meleagris gallopavo* XP_003203573.1
4	1.6	1.6	0.8		99.5	98.4	98.1	98.7	98.7	97.3	97.0	97.0	97.0	96.5	96.2	84.4	87.4	4	*Coturnix japonica* XP_015707880.1
5	2.7	1.6	0.5	0.5		98.9	98.7	99.2	99.2	97.8	97.6	97.6	97.6	97.0	96.8	84.1	87.1	5	*Phasianus colchicu* s XP_031468291.1
6	1.6	2.7	1.1	1.6	1.1		99.2	99.7	99.7	97.3	96.5	96.5	96.5	96.0	96.0	84.1	86.3	6	*Tympanuchus pallidicinctus* XP_052526080.1
7	2.5	2.5	1.4	1.9	1.4	0.8		99.5	99.5	97.6	96.5	96.5	96.5	96.0	96.0	84.1	86.0	7	*Centrocercus urophasianus* XP_042665065.1
8	2.5	2.5	0.8	1.4	0.8	0.3	0.5		100.0	97.6	96.8	96.8	96.8	96.2	96.2	84.1	86.3	8	*Lagopus muta* XP_048796186
9	2.5	2.5	0.8	1.4	0.8	0.3	0.5	0.0		97.6	96.8	96.8	96.8	96.2	96.2	84.1	86.3	9	*Lagopus leucura* XP_042718429.1
10	3.0	3.0	2.2	2.7	2.2	2.7	2.5	2.5	2.5		96.2	96.2	96.0	95.7	95.7	83.9	85.5	10	*Numida meleagris* XP_021238017.1
11	3.6	3.6	3.0	3.0	2.5	3.6	3.6	3.3	3.3	3.9		100.0	100.0	98.9	98.1	84.4	87.6	11	*Anas platyrhynchos* XP_027322867.1
12	3.6	3.6	3.0	3.0	2.5	3.6	3.6	3.3	3.3	3.9	0.0		100.0	98.9	98.1	84.4	87.6	12	*Aythya fuligula* XP_032047136.1
13	3.6	3.6	3.0	3.0	2.5	3.6	3.6	3.3	3.3	4.1	0.0	0.0		98.9	98.1	84.1	87.6	13	*Cygnus olor* XP_040400244.1
14	4.1	4.1	3.6	3.6	3.0	4.1	4.1	3.9	3.9	4.4	1.1	1.1	1.1		98.7	84.7	87.6	14	*Pezoporus wallicus* XP_057255016.1
15	4.4	4.4	3.6	3.9	3.3	4.1	4.1	3.9	3.9	4.4	1.9	1.9	1.9	1.4		84.1	86.8	15	*Calidris pugnax* XP_014813843.1
16	17.9	17.9	17.9	17.5	17.9	17.9	17.9	17.9	17.9	18.2	17.6	17.6	17.9	17.2	17.9		91.1	16	*Mus musculus* NP_001347681.2
17	15.2	15.2	14.5	13.9	14.2	15.2	15.5	15.2	15.2	16.2	13.6	13.6	13.5	13.5	14.5	9.5		17	*Homo sapiens* NP_001280108.1
	1	2	3	4	5	6	7	8	9	10	11	13	13	14	15	16	17		

Figure S4: Amino acid sequence identity of PRSS23 between Wuding chicken and other species

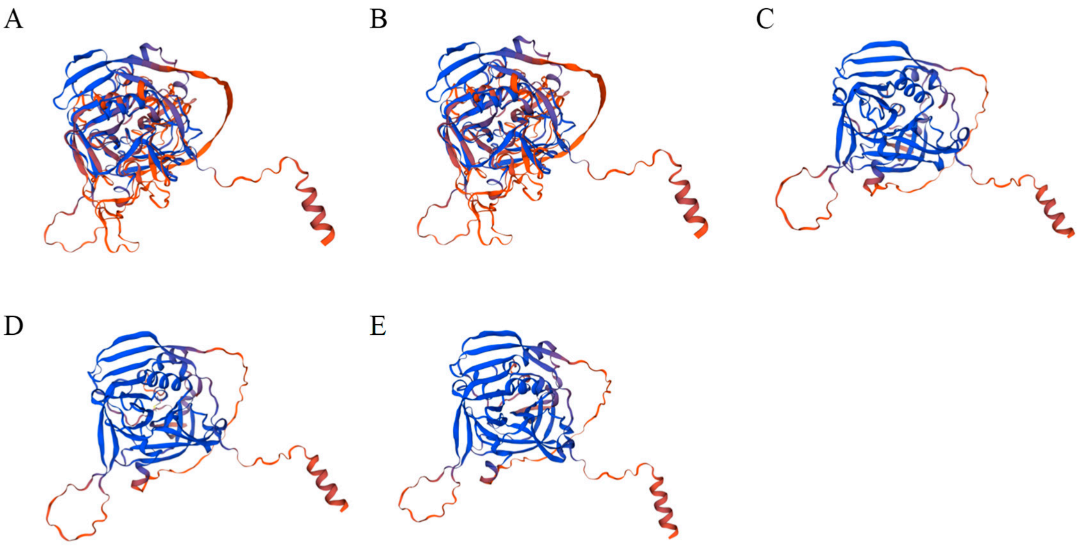

Figure S5. The tertiary structure of PRSS23 mature peptides in Wuding chicken and other pheasant species； Wuding chicken (A); *Gallus gallus* (B); *Meleagris gallopavo* (C); Coturnix japonica (D); *Phasianus colchicus* (E)
